# Antecedents of Knowledge Hiding and Their Impact on Organizational Performance

**DOI:** 10.3389/fpsyg.2021.796976

**Published:** 2021-12-20

**Authors:** Junqi Wen, Ruijun Ma

**Affiliations:** ^1^School of Labor and Human Resources, Renmin University of China, Beijing, China; ^2^Beijing Docvit Law Firm, Tianjin, China

**Keywords:** knowledge hiding, lack of rewards, knowledge sharing, internal competition, psychological entitlement, social status, organizational performance

## Abstract

Research on knowledge management has rapidly increased in the last decade, leaving a huge gap on how, why, and what triggers knowledge hiding in inter-organizational setups. Furthermore, the fostering factors for knowledge sharing have also remained unexplored because the employees in an organization are unwilling to share their knowledge with others for several reasons. The current study has attempted to explore the reasons that make employees hide their knowledge from other employees in order to excel. The individual factors considered in this study that make employees hide their knowledge are the lack of rewards for knowledge sharing, internal competition, and psychological entitlement. Furthermore, the interesting consequent factor of knowledge hiding in this study was found to be significant. The moderating role of employees’ social status has a significant moderating effect on the relationship between knowledge-hiding behavior and organizational performance. The population of the study was the managerial employees of financial institutions of China and the sample size taken in his study was 446 *via* convenient sampling technique. The independent factors in this study found significant results of knowledge-hiding behavior, thus approving the mediating role of knowledge hiding in the organizational performance of the financial institutions of China. The software used in this study for the data analysis was smart PLS and the technique used was partial least square SEM for the measurement of the hypothesis of the study. The study’s findings also have certain implications for policymaking in financial institutions that may hinder knowledge hiding practices and support the uninterrupted flow of knowledge among employees.

## Introduction

Organizational performance is directly related to the performance of employees. Sometimes, workers are so good in their working ability, they are compensated. Often, they work well, but they do not have the opportunity to showcase their talent. The ability to work in an organization is not the only indicator of the success of the organization. The major part of the success of organizations comes through the knowledge of workers and stakeholders. Knowledge sharing is an important component of success at the organizational level. A lot of work has been done in the past to identify the impact of this knowledge sharing.

Contrary to knowledge sharing, knowledge hiding may sometimes be useful for an organization to improve its internal competitive ability. In the business world, knowledge sharing has received a lot of attention. When reading organizational theory literature, one cannot help but notice a shift in emphasis from more meaningful and measurable elements of business to softer aspects, with academicians and researchers alike paying growing attention to concepts like creative thinking, quickness, and understanding.

This is an understandable reaction to the constant change and unpredictability of the global business environment, which renders traditional tactics useless and forces firms to adopt new approaches that are more appropriate for the times. Most research claims that knowledge sharing among the management of purchasing and providing enterprises is critical for organizational success (Pérez-Salazar et al., 2019). Existing marketing literature also claims that cross-organizational sharing of knowledge is the glue that can tie companies together, as good knowledge sharing enables companies to share important information. Knowledge sharing is a vital relational ability that can result in a slew of benefits for supplier relationship companies and the eventual success of buyer-supplier relationships. A successful supply chain outcome is also linked to good knowledge sharing among supply chain member organizations. Although the necessity of information transmission between organizations has long been recognized, some academics claim that knowledge hiding among employees across enterprises can hurt a company’s capacity to compete and develop and inevitably lead to client relationship breakdown (Avotra et al., 2021a,b).

Managers at all levels of a company can participate in knowledge concealment for a variety of reasons. If there is a trust gap between them, they may first hide information from one other. They may develop a trust deficit as a result of a lack of personal relationship or friendship between them, or if they believe their colleague lacks the necessary expertise to complete the task, mangers might also hide knowledge if their company’s culture discourages it. Owusu Kwateng et al. (2021) claim that managers in different companies can intentionally keep information from one another, which has the ability to ruin buyer–supplier interactions. However, research on buyer–supplier relationships is limited and difficult to arrange. Knowledge hiding is a fascinating notion with clear negative implications for businesses, as it inhibits innovation, hinders teamwork and collaboration, and eventually impairs the organization’s performance (Yingfei et al., 2021).

To begin, we will define and delineate the meaning of the fundamental phrase, and evaluate what is meant by knowledge. Knowledge is a notion in philosophy that encompasses the ways of comprehending what you know, how you came to know it, and what it means. Whereas the significance and description of knowledge would seem to be a point of contention among epistemologists, it is widely accepted, for this study, as theoretical and/or practical familiarity with and understanding of a subject, such as skills, facts, and objects, and as true belief with justification. On the other hand, knowledge management is crucial for long-term growth and market success (Sukumaran and Lanke, 2021). However, there are few studies on the predictors and repercussions of information concealment in the workplace, and the function of knowledge hiders and seekers in this setting is mostly unknown. Knowledge is seen as a vital resource for an organization’s long-term success and viability. Organizations rely on their merchandise project team to create innovative brands, which necessitates knowledge capitalization across the firm (An et al., 2021). Members of the project team increase their performance by effectively sharing knowledge and promoting creativity (Muhammed and Zaim, 2020).

Employees have been found to share their knowledge with others in some firms proactively. These organizations have fared better in generating new ideas; a permissive knowledge-sharing environment is a requirement for this. However, there appears to be very little (albeit urgently required) research on how information concealment affects outcomes. The purposeful and deliberate endeavor by employees of businesses to hide their expertise and vital understanding from their colleagues is referred to as knowledge hiding. Employees’ attempts to conceal knowledge are common whenever a development team is working on a new product. When staff are discovered to be primarily engaged in knowledge hiding, the company becomes a knowledge-hiding company. The performance of a development team is measured by how closely they comply to the organization’s objectives, which include performance, cost, and timeliness. Employees’ creative ideas have an impact on the success of the team’s performance.

This is damaged when information hiders are discovered hiding their knowledge, which can subsequently become the core cause of an organization’s downfall. People are driven to conceal knowledge if there is mutual distrust, particularly if this motivation is strong, which is harmful to the organization’s effectiveness (Ali et al., 2021; Xiaolong et al., 2021). Nevertheless, very little research has been done to find a link between knowledge hiding and organizational performance. Since many spend time researching how knowledge sharing affects organizational performance, the potential effect of employees hiding knowledge and how it can limit organizational success in the future remains a study gap (Cegarra-Navarro and Martelo-Landroguez, 2020; Chatterjee et al., 2021). “A purposeful effort by a person to conceal or hide the knowledge that has been requested by another person” is how knowledge hiding is defined. Much research has been conducted on the causes and implications of knowledge concealment since the construct was established in 2012. Organizations, partnerships, and employees are all affected by knowledge concealment. Decreased levels of creativity and innovative work behavior and lower individual performance have been related to it.

Knowledge hiding has been connected to an increase in mutual distrust and a decline in interpersonal relationships. Recent research has also looked into the origins of knowledge hiding. In situations of strong distrust, rivalry, or perceived organizational politics, knowledge concealment has been proven to rise. On the other hand, knowledge hiding is decreased in environments where there is reciprocal social exchange, a mastery climate, or when individuals have high degrees of showing goal-oriented or pro-social motivation (Rhee and Choi, 2017). Knowledge hiding is a multifaceted concept with three dimensions. Accordingly, rationalized knowledge hiding is the least deceiving and makes reference to how a hider gives a clear picture as to why the information will not be forthcoming; evasive hiding occurs when the hider provides incorrect or partial information or a misleading promise of a more complete explanation in the future; and playing dumb refers to instances where the hider feigns ignorance to avoid providing the information (Connelly et al., 2019).

Our research is equipped with certain objectives. An intensive literature survey is used to build the research’s theoretical underpinning, which culminates in constructing a set of hypotheses and a conceptual model. Following that, the research technique is described, followed by the analysis and presentation of the findings (hypotheses testing). Following that, the necessary debate and explanation of the research’s theoretical contribution and practical ramifications occur. This paper concludes with conclusions, limits, and recommendations for future research. The following objectives were outlined: (1) To determine the contributors of knowledge hiding and their significance, (2) to evaluate the mediating role of knowledge-hiding behavior, (3) to analyze the determinants of organizational performance, and (4) to assess the moderating role of social cost on organizational performance.

## Review of Literature

### Impact of Lack of Knowledge Sharing Reward on Knowledge Hiding

Rewards could include monetary incentives like bonuses, non-monetary prizes like restaurant gift vouchers, and non-monetary honors like praise and public recognition. Intrinsic rewards, such as the pleasure gained from completing the task, are also possible. Gerhart (2017) discovered five key characteristics of organizational incentive systems that effectively motivate people to engage in the desired activities. These characteristics include, but are not limited to, perceived reward fairness, employees establishing tough goals to earn appealing benefits, and methods that ensure employees have high self-efficacy in doing the tasks. Two key prerequisites for reward systems to achieve these criteria and be effective are that the incentive giver can witness or record the target behavior and assess its worth. Due to the three kinds of hurdles (individual/personal, organizational, and technical) for knowledge sharing, which include aspects such as apprehension about failures, knowledge base compatibility, cost of imitation and its inherent fuzziness, cross-cultural barriers, and knowledge hoarding, rewards are considered an important element in facilitating knowledge sharing and learning.

The function of rewards in knowledge management inside businesses has been the subject of recent research (Pittino et al., 2018; Lyu et al., 2020). For example, by looking at the design of a knowledge-sharing incentive system and evaluating the effect of an individual-based vs. a group-based reward system, researchers discovered that normative incentives combined with hedonic motivation in extrinsic incentives could increase information sharing. They investigated the impacts of both internal and extrinsic incentives on information sharing, demonstrating that in a knowledge management system, reputation feedback promotes successful knowledge sharing (KMS) (Nguyen et al., 2019). Knowledge hiding persists in firms, despite managers investments in facilitating knowledge exchange. Existing research sheds light on the causes and effects of information concealment from the perspective of the hider (Wang et al., 2019). There are several reasons behind knowledge hiding, but the most possible reason is the employees’ lack of knowledge-sharing rewards. Rewards for knowledge sharing surely have a strong impact on not hiding knowledge for organizational success. This led us to hypothesize the following.

H_1_: Lack of knowledge sharing reward has an impact on knowledge hiding.

### Impact of Internal Competition on Knowledge Hiding

Organizational performance is extremely competitive, and corporations employ intra-organizational competitiveness to distinguish between successful and unsuccessful employees. Competition is described as rivalry in the sale of goods and services, in which rivals compete for similar resources and incentives that are too limited to be shared equally. Competition acts as a kind of goal-setting in which the competence of contestants equates to the individual’s performance targets or standards. External competition and organizational performance have received a lot of attention in the for-profit world but less so in the non-profit world. The conception of competition within a non-profit organization suggests a different form of competition: internal competition. Internal competition for time and energy spent on different objectives occurs between departments, between individuals, and even among individuals in the non-profit endemic environment of restricted resources (Mottner and Ford, 2008). Internal competition takes the form of internal conflicts over goals and the means to achieve them.

Internal competition is a powerful motivator for increasing sales because it encourages staff to set higher targets than they would otherwise (Kalra et al., 2021). Internal competition, unfortunately, leads employees to see their coworkers as true competitors. Even so, they are contending for a prize that only a select few will receive. A competent marketer protects their advantages from the competition. Market expertise is one of their most substantial advantages. Employees hesitate to share valuable market information with their coworkers to surpass the competition. When a sales organization holds a sales contest, it creates a competitive environment where employees compete for a limited number of prizes. Competitor autonomy is a concept rooted in the economic theory of perfect competition, in which protected knowledge that gives a competitor employee an advantage is shielded from a competitor. Sharing market knowledge with coworkers erodes an employee’s competitive advantage as well as the foundation upon which the employee’s influence inside the company is built (Anaza and Nowlin, 2017). So, there is a strong background for knowledge hiding among the workers due to the internal competition, which enables us to formulate the following hypothesis for analyzing the impact of internal competition on knowledge hiding.

H_2_: Internal competition has an impact on knowledge hiding.

### Impact of Psychological Entitlement on Knowledge Hiding

Knowledge-sharing research has flourished in recent decades as a result of changing trends and the integration of knowledge and talent management in the modern organizational setup. Knowledge hiding has gone unexplored; furthermore, initiatives to promote expertise sharing have stalled because employees are afraid to share their knowledge for a variety of reasons. The relationship between psychological entitlement and knowledge-hiding behavior was explored using psychological ownership and social exchange theory. A widespread and stable belief that one deserves and is entitled to more than others is psychological entitlement. Individuals who are psychologically entitled are more prone to prefer themselves and believe they are worthy of incentives and recognition. Knowledge sharing and hiding are two opposites whereas knowledge ownership is a contentious issue in the workplace, and it provides a fertile ground for potential conflict between employees and employers (Brown et al., 2014; Harvey et al., 2014). According to the researchers, organizations’ desire to “own what you know” might lead to such confrontations with their personnel working for them.

In the context of this study, ownership psychology refers to the sensation of being mentally attached to an object. Employees’ feelings of knowledge ownership and possession are thus defined by psychological ownership of knowledge (Han et al., 2015). As a result, knowledge may be shared or kept hidden. Ownership-driven knowledge concealment happens due to one of two factors: (1) An overvaluation of information or (2) the fear of losing control. Based on the psychological ownership of knowledge paradigm, we argue that stronger psychological entitlement predicts increased knowledge-concealment behavior among bank employees. Individuals frequently devote money, time, and mental energy to acquiring knowledge through formal education, experience, and training, which is one potential explanation. Knowledge hiding comes through a self-satisfaction of a psychological perspective. Because sharing is the same as transferring ownership, persons who have a strong feeling of psychological knowledge ownership are more likely to have low knowledge-sharing incentives and high knowledge-concealment behavior. This develops a strong relationship between psychological entitlement and knowledge hiding, so the following hypothesis was developed based on the aforementioned arguments.

H_3_: Psychological entitlement has an impact on knowledge hiding.

### Impact of Knowledge Hiding on Organizational Performance

Since today’s businesses operate in such a complicated and volatile environment, organizational knowledge management has become important. Sharing knowledge is necessary for a good organization; it aids in achieving a competitive advantage and promotes a sharing attitude by assisting others with various activities that take place in the workplace. Employees’ knowledge refers to the information or skills needed to complete organizational duties. Employees frequently engage in information concealment practices, squandering organizations’ efforts to ensure knowledge sharing (Hamza et al., 2021). An intentional attempt by an individual to conceal or hide the knowledge that a coworker has sought is known as knowledge hiding. Because information concealment is not the polar opposite of knowledge sharing, it is critical to comprehend the impact of knowledge hiding in businesses. The performance of employees suffers as a result of knowledge hiding. Employees develop a culture of distrust and hostility, which reduces corporate affiliation and increases employee turnover. As a result, information hiding is harmful to both employees and the organization. Furthermore, firms will become increasingly knowledge-intensive.

With a more diverse workforce, a strong framework is required to remove hidden motives or matters related to morals or cultural barriers, encouraging solidarity and cooperation. Given the problem’s gravity, management has had little success in preventing knowledge concealment (Anand and Hassan, 2019). As previously stated, knowledge hiding happens when members conceal or hide their exclusive knowledge (e.g., achievements, expertise, and know-how) that is required by others. When viewed in this light, knowledge concealment is distinct from many other unproductive knowledge behaviors. The concept of knowledge hiding, for starters, refers to the purposeful attempt of knowledge owners to hide their knowledge.

On the other hand, partial knowledge sharing entails objective actions that might influence unforeseen circumstances and communication channels. Researchers said knowledge isolated people from organizational knowledge transfer and integrating social networks (Phelps et al., 2012). Since team members cannot get much valuable information from their teammates, they have to spend a lot more time looking for and integrating information from other places. As a result of the knowledge transfer issues created by knowledge-hiding behavior, the project’s completion time is extended and the time to market is slowed (Fixson and Marion, 2012). Since knowledge hiding has been found to have a large impact on organizational performance in the past, we formulated the following hypothesis in this regard.

H_4_: Knowledge hiding has an impact on organizational performance.

### Mediating Role of Knowledge Hiding Among Various Factors

Knowledge hiding is a term that encompasses three main methods used by individuals to keep information hidden from others. These approaches include: (1) Justifying hiding, in which a collection of descriptive assertions or justifications are offered to substantiate the grounds for such knowledge source’s inaccessibility; (2) deceptive hiding, in which people postpone or disclose less than is truly required for the other person; and (3) playing dumb, in which people pretend to be oblivious of information or knowledge.

For both the hider and the seeker, evasive hiding has the most unfavorable consequences. Because information hiding causes significant harm, it is critical for managers to keep track of the reasons of knowledge concealment in the workplace (Argote and Fahrenkopf, 2016). Understanding the causes of knowledge concealment is critical, as it allows professionals to mitigate harmful repercussions. Recent studies on information hiding have looked into a range of reasons employees hide their knowledge (Connelly and Zweig, 2015). Employees are expected to share their knowledge with coworkers in order for firms to prosper and remain competitive. Although corporations make a concerted effort to encourage individuals to share knowledge, the success of such efforts is contingent on the employees’ willingness and intent to share knowledge and numerous events that occur within the organization.

Knowledge is a valuable resource and knowledge sharing is dependent on individuals deciding with whom, when, and why to share it; certain factors such as internal competition, psychological entitlement, lack of knowledge sharing rewards, and a lack of organizational culture come into play to explain why people may not share knowledge, even if it is valuable (Lanke, 2018; Nugroho, 2018; Gagné et al., 2019). Employees can use knowledge to advance their business status, but they can also hide it to improve their value and become essential. Various researchers have looked into the factors facilitating knowledge sharing at both personal and organizational levels (Nugroho, 2018). Many papers have been published in recent years looking into the hurdles to knowledge sharing. Such restrictions, which include knowledge hiding, knowledge hoarding, and knowledge withholding, are defined as actions taken by people who refuse to share their information.

In addition, there has been a growing interest in why people in organizations hide, withhold, or hoard knowledge from others. As a result, both scholars and practitioners have begun to pay more attention to the concept of knowledge concealment (Connelly and Zweig, 2015; Škerlavaj et al., 2018; Connelly et al., 2019). According to some experts, knowledge hiding is not the same as knowledge hoarding, as knowledge hiding is simply the inability to share knowledge. This may occur because people are unaware of the need for knowledge among others. Concealment might thus be deliberate, contextual, or affected by both internally and externally motivational variables. Others, on the other hand, use the term knowledge hiding interchangeably with knowledge hoarding (de Geofroy and Evans, 2017; Anand and Hassan, 2019). Furthermore, the terms information hiding and knowledge hoarding are frequently utilized to characterize non-sharing behavior. Knowledge hiding and storing, according to another study, are hurdles to sharing knowledge and might be classified as knowledge withholding behaviors (Holten et al., 2016).

Connelly’s view of information concealment at the individual level is based on the idea that there should be a request from a knowledge seeker that the knowledge provider does not meet. Sharing knowledge and hiding, on the other hand, can occur on different levels. For example, according to Anand, an individual can share or hide his or her knowledge depending on his or her willingness and intention from one individual to another, an individual to a group, a group/organization to an individual, or a group/organization to another group/organization. At a collective level, factors include group culture, group task and group characteristics, group beliefs, organizational resources, negative coworker interactions and interpersonal conflicts, organizational climate, poor organizational culture, and rewards (Holten et al., 2016). At an organizational level, factors include workplace incivility, organizational climate, poor organizational culture, and rewards (Arshad and Ismail, 2018). Some people may consider knowledge hiding to be a constructive act. Connelly, for example, claims that knowledge concealment is not bad because it can be modified by a pro-social motive. Hiders’ and targets’ interpersonal ties can be strengthened through rationalized concealment, for example.

However, research focuses more on understanding the negative implications of knowledge concealment and, in particular, on getting companies to focus on the following two fundamental questions: Why do employees keep their information hidden? What can businesses do to prevent knowledge from being hidden in the workplace? (Xiong et al., 2021). Given the implications, managers have not been successful in managing knowledge-hiding behavior, so therefore contribute to current discussions about knowledge concealment by suggesting numerous circumstances that may lead to knowledge-hiding behavior. The researchers aim to demonstrate knowledge concealment as a distinct construct, despite certain overlaps between the notions. This effectively expands the knowledge transfer span. Some other contrasts to be drawn are between knowledge concealment and knowledge dissemination. According to the scientists, these characteristics are not opposed but rather completely different.

Knowledge concealment and a lack of knowledge sharing in an organization are not the same thing when it comes to motivation. Knowledge concealment might be the consequence of a desire to conform to societal norms, or it can be a means to an end or the consequence of carelessness. Lack of knowledge-sharing reward is frequently the result of a scarcity of genuine meaningful information. Knowledge concealment is also distinct from ineffective workplace activities. Individuals who initiate knowledge concealment may not always mean to hurt others, unlike counterproductive activities that have a negative impact on other people and thus the organization. In the former, the target is always an individual, whereas in the latter, both the organization and individuals may be victims (Kumar Jha and Varkkey, 2018). Knowledge concealment may appear to be similar to social undermining, which is designed to obscure a worker’s abilities and ruin their reputation, but it is distinct since, as previously stated, the goal of knowledge concealment is never to harm others. Workplace incivility can be blamed for rude, uncourteous behavior, but knowledge concealment is not the same.

There are times where knowledge is hidden without being disrespectful (Issac and Baral, 2018). The mediating role of knowledge sharing has been studied many times in the past but the mediating role of knowledge hiding needs to be understood in this modern era. Very few research projects have been completed on mediating factors like the study by Alnaimi and Rjoub (2021). So, there was a dire need to study the mediating role of knowledge hiding on organizational performance utilizing different variables. This research, based on the supporting literature about knowledge hiding, hypothesized the following for evaluating the organizational performance with the mediating role of knowledge hiding.

H_5_: Knowledge hiding mediates the relationship of lack of knowledge-sharing rewards and organizational performance.

H_6_: Knowledge hiding mediates the relationship of internal competition and organizational performance.

H_7_: Knowledge hiding mediates the relationship of psychological entitlement and organizational performance.

### Moderating Role of Social Status of Employees

Social status refers to a person’s honor, prestige, and influence as a result of his or her possession of desirable talents that can help them achieve their goals in a specific social situation. An individual’s social status is also attributed to someone who can improve group performance by surrendering his or her own resources for the sake of others. In the current research approach, social status is extremely significant since it impacts positive interactions by boosting an individual’s confidence and ease in forging social partnerships in his or her favor based on the unique resources he or she holds (Lawler and Thye, 1999). Because social status suggests an individual’s ownership of task-relevant resources, knowledge receiving also denotes the knowledge giver’s social standing in order to appraise the reliability, value, and validity of the supplied knowledge. As a result, the consequences of knowledge management are determined by one’s social status.

Firstly, we believe that employees’ social position will amplify the effect of knowledge exchange on their innovation. High-status individuals’ knowledge and know-how are highly valued, well accepted, and frequently reciprocated by others who see such knowledge as useful and trustworthy resources for achieving their objectives (Groysberg et al., 2010). Coworkers value and respond favorably to high-status individuals sharing their information, and they reciprocate by offering their own knowledge and providing various types of aid to beneficent high-status members. High-status individuals are well positioned to gain from the social exchange benefits of knowledge sharing by getting various forms of reciprocating returns from others in order to boost their inventiveness. Secondly, we suppose such high-status individuals want to keep their information hidden. In that case, the negative consequences can be severe since others perceive them as a crucial source of high-quality knowledge and expect them to contribute their expertise to the attainment of a common goal.

Whenever the expectation (or, in some ways, social responsibility) is not met, the assignment of such an action to egocentric, free-riding drive becomes prominent and dangerous for high-status individuals (Weiss and Morrison, 2019). On the other hand, individuals are less concerned about low-status members, with no valuable information for the community, hiding their knowledge. Therefore, choosing this conscience knowledge-handling method may not result in harsh reactions from others. As a result, because of the severe social repercussions (e.g., negative reciprocity or punishment) when high-status members withdraw from knowledge exchange, the negative effect of knowledge concealment on creativity may be particularly salient for them. The moderating role of social status (Rhee and Choi, 2017) has minutely been studied so far but it has a lot of potential for future research. So, keeping in view the substantial moderating role of social status, the following was hypothesized.

H_8_: Social status of employees moderates the relationship of knowledge hiding and organizational performance.

**FIGURE 1:**
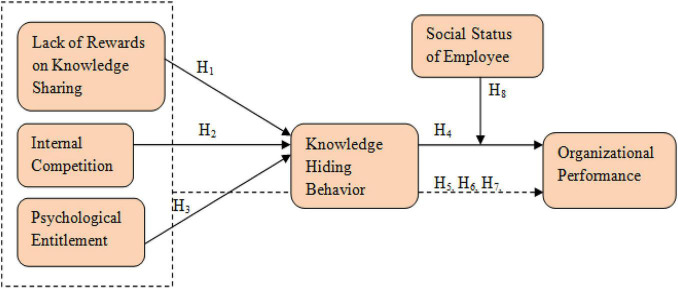
Conceptual model.

Based upon the literature review, this research was designed and the following conceptual framework was developed. The research revolves around this concept (see Figure 1).

## Methodology

Quantitative techniques for data analysis have been used in this study. Different hypotheses have been derived from the literature, supported by different theories of management leading to certain behaviors, hence the following deductive approach was used to reach specific behaviors mentioned in the conceptual framework of the study. The most widely used method for data collection in management and business studies is a survey. The current study has also undertaken this method of survey to authenticate the proposed conceptual framework regarding the knowledge-hiding behavior. Moreover, surveys have been considered an effective option to get hold of respondents’ perception to measure the causal effects of different variables (Ahbabi et al., 2019). Furthermore, surveys also allow the collection of data from relatively larger samples of the population that allow the generalizability of findings. The demographic data collected were analyzed, then preliminary reliability and validities were checked before final measurement of relationships using smart PLS 3.

### Instrument Development

The underlying items of each variable were organized in a survey questionnaire. The survey questionnaire was developed on a five-point Likert scale with 5 being the highest rate of agreement to the statement and 1 being the lowest agreement rate. The questionnaire comprised 32 questions over seven sections in total and was adapted from the respective literature. The first section covered the demography of the respondents, the second section was about the organizational performance containing eight items (Ahbabi et al., 2019), the third section was about the lack of rewards on knowledge sharing containing six items (Anaza and Nowlin, 2017), internal competition with six items (Anaza and Nowlin, 2017), psychological entitlement with four items (Alnaimi and Rjoub, 2021), knowledge-hiding behavior (Černe et al., 2017), and social status of employees with four items (Rhee and Choi, 2017). The items adapted had been face and content-validated from five managers of the financial service industry of China and an academic expert in the knowledge management field to review the designed survey instrument, as the population of the study was also managers serving in the financial industry in China. The suggestions were found appropriate according to the requirement of the study and then the questionnaire was surveyed electronically by taking prior consent from the organizations keeping the identity of the respondents anonymous. We received 505 responses, of which 446 were used for data analysis which made the response rate 88.3%. The sample size for this study was 446 using convenient sampling. The details for the demographic particulars are listed in Table 1. This included the responses for age, gender, education, and managerial position.

**TABLE 1:** Demographics of the respondents.

Age	Frequency	Percentage
<20	84	18.83
21–29	128	28.69
30–39	84	18.83
40–49	35	7.84
49>	115	25.78
**Gender**		
Male	245	54.93
Female	201	45.06
**Education**		
Bachelor	68	15.24
Masters	158	35.42
Doctorate	65	14.57
Others	154	34.52
**Position**		
Entry level	101	22.64
Middle level	158	35.42
Senior level	187	41.92

*N = 446.*

### Data Analysis

**FIGURE 2:**
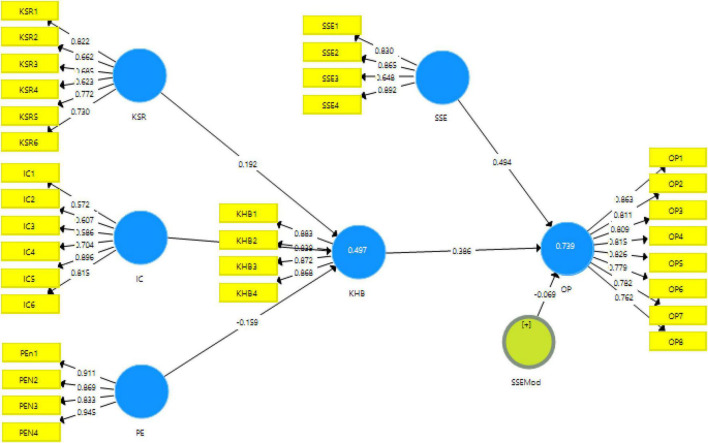
Measurement model algorithm.

Recent literature has been using structural equation modeling as a substitute for regression analysis (Nawaz et al., 2019). The current study has also used smart PLS 3.3 for the preliminary validation and measurement of hypotheses for this study. This has been done in two steps. In the first step which is the measurement model, the data are validated and reliability of data is tested. While in the second step, which is structural equation modeling, hypothesis testing is carried out with the help of a bootstrapping algorithm with 500 iterations (Henseler et al., 2015). The measurement model for the study can be seen in Figure 2. The tables for the validities and reliabilities can be seen in Tables 2–4.

**TABLE 2:** Construct reliabilities and AVE.

Constructs	Code	FD	α	CR	AVE
Lack of rewards on knowledge sharing			0.839	0.864	0.517
	KSR1	0.822			
	KSR2	0.662			
	KSR3	0.685			
	KSR4	0.623			
	KSR5	0.772			
	KSR6	0.730			
Internal competition			0.845	0.854	0.501
	IC1	0.572			
	IC2	0.607			
	IC3	0.586			
	IC4	0.704			
	IC5	0.896			
	IC6	0.815			
Psychological entitlement			0.912	0.939	0.793
	PEN2	0.869			
	PEN3	0.833			
	PEN4	0.945			
	PEN1	0.911			
Knowledge-hiding behavior			0.888	0.923	0.749
	KHB1	0.883			
	KHB2	0.839			
	KHB3	0.872			
	KHB4	0.868			
Social status of employee			0.830	0.886	0.663
	SSE1	0.830			
	SSE2	0.865			
	SSE3	0.648			
	SSE4	0.892			
Organizational performance			0.923	0.937	0.650
	OP1	0.863			
	OP2	0.811			
	OP3	0.809			
	OP4	0.815			
	OP5	0.826			
	OP6	0.779			
	OP7	0.782			
	OP8	0.762			

*N = 446; FD, factor loading; AVE, average variance extracted; CR, composite reliability.*

**TABLE 3:** HTMT ratio.

	IC	KHB	KSR	OP	PE	SSE
IC						
KHB	0.600					
KSR	0.821	0.536				
OP	0.637	0.855	0.577			
PE	0.652	0.213	0.584	0.149		
SSE	0.881	0.784	0.691	0.878	0.362	

*N = 446; IC, internal competition; KHB, knowledge-hiding behavior; KSR, lack of rewards on knowledge hiding; OP, organizational performance; PE, psychological entitlement; SSE, social status of employee.*

**TABLE 4:** Fornell and Larcker criterion.

	IC	KHB	KSR	OP	PE	SSE
IC	0.708					
KHB	0.680	0.866				
KSR	0.707	0.567	0.719			
OP	0.791	0.782	0.632	0.806		
PE	0.452	0.192	0.378	0.137	0.890	
SSE	0.651	0.695	0.613	0.796	0.287	0.814

*N = 446; IC, internal competition; KHB, knowledge-hiding behavior; KSR, lack of rewards on knowledge hiding; OP, organizational performance; PE, psychological entitlement; SSE, social status of employee.*

**FIGURE 3:**
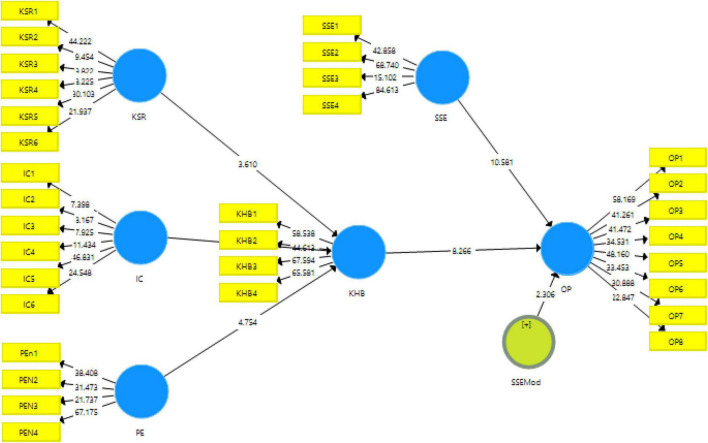
Structural model algorithm.

One of the most commonly used techniques for validity is factor loading. The measurement model algorithm obtained from smart PLS 3 gives the results for factor loadings as well. The factor loadings obtained in this study are all above the threshold of 0.5 defined in the literature. The minimum factor loading of the current study is 0.572 which is well above the threshold, as can be seen in Table 2 and Figure 3. Similarly, regarding the reliabilities, i.e., Cronbach alpha reliability and composite reliability, the cut off value mentioned in literature is 0.7, however, the obtained reliabilities in this research for the data are above 0.8 which is very good. Furthermore, regarding the average variance extracted (AVE), reported in Table 2, the lowest acceptable value given in previous research is 0.5 (Franke and Sarstedt, 2019). The current study meets these criteria of AVE and the values reported in this study are all above 0.50 which validate the data in this study.

The data acquired from the analysis using Smart PLS were further validated with hetero trait mono trait, i.e., HTMT ratio, and Fornell and Larcker criterion. For valid data, according to literature, values obtained in the HTMT ratio should be less than 0.9 (Henseler et al., 2015). In this study, the reported values of HTMT ratio are valid making the data valid. Additionally, for Fornell and Larcker criteria, according to Franke and Sarstedt (2019) the topmost values in each column should be among the highest values. Likewise, in the described values in Table 4 for Fornell and Larcker criteria, the values on the top of the column are among the highest values of each column thus meeting the validity for the study.

Furthermore, in the second stage of smart PLS, the data were analyzed for the acceptance or rejection of the hypotheses *via* the structural model. Through this model the hypotheses were checked for the significance using t-statistics, beta values, *p*-values, and overall adjusted R^2^ values of the dependent variables. These results are reported in Table 5 based on bootstrapping. There were a total of eight hypotheses developed in this study based on the gaps obtained from the literature. The first hypothesis was H_1_: Lack of knowledge-sharing reward has an impact on knowledge hiding. This hypothesis was accepted at a 1% significance level with t-statistic = 3.610. The second hypothesis regarding the internal competition (H_2_: Internal competition has an impact on knowledge hiding) was also supported by the results obtained at a 1% significance level with t-statistic = 12.911. Similarly, the third hypothesis was also supported by the results (H_3_: Psychological entitlement has an impact on knowledge hiding) but it found a negative effect on knowledge hiding (t-statistic = 4.754 and *p*-value < 0.005). Similarly, H_7_: Knowledge hiding mediates the relationship of psychological entitlement and organizational performance was also found to have negative but significant results (t-statistic = 3.946 and *p*-value < 0.000). Regarding H_4_: Knowledge hiding has an impact on organizational performance, the results were also found to be significant at a 1% *p*-value with t-statistic = 8.266 hence approving the hypothesis. H_5_ and H_6_ were also approved at a *p* < 0.005 significance level with t-statistics = 3.168 and 6.784, respectively. As for the last hypothesis of the study, H_8_, it was found to have negatively moderated the relationship of knowledge hiding and organizational performance (t-statistic = 2.306 and *p*-value < 0.05). Results of the study hypotheses can be seen in Table 5.

**TABLE 5:** Results for structural model.

Paths	H	O	M	SD	T-stats	*P*-value	Results
KSR→KHB	H_1_	0.192	0.196	0.053	3.610	0.000***	Accepted
IC→KHB	H_2_	0.615	0.612	0.048	12.911	0.000***	Accepted
PE→KHB	H_3_	−0.159	−0.153	0.033	4.754	0.000***	Accepted
KHB→OP	H_4_	0.386	0.380	0.047	8.266	0.000***	Accepted
KSR→KHB→OP	H_5_	0.074	0.075	0.023	3.168	0.002**	Accepted
IC→KHB→OP	H_6_	0.238	0.233	0.035	6.784	0.000***	Accepted
PE→KHB→OP	H_7_	−0.061	−0.058	0.016	3.946	0.000***	Accepted
SSE→Mod→OP	H_8_	−0.069	−0.073	0.030	2.306	0.021*	Accepted

N = 446; H, hypotheses; O, original sample; M, sample mean; SD, standard deviation. ***p < 0.0005, **p < 0.005, and *p < 0.05. IC, internal competition; KHB, knowledge-hiding behavior; KSR, lack of rewards on knowledge hiding; OP, organizational performance; PE, psychological entitlement; SSE, social status of employee; knowledge-hiding behavior adjusted R^2^ = 0.494, organizational performance adjusted R^2^ = 0.737.

## Discussion

The current study aimed to assess the antecedents of knowledge-hiding behaviors and their consequent effect on organizational performance in the financial service industry in China. Moreover, it has also addressed the moderating role of social status of employees in this whole scenario. Knowledge-hiding behavior is a very under-studied variable in organizations which creates opportunities for new studies in this online and work-from-home era. The hypotheses of the study were firstly analyzed using the demography of the respondents. The education of the respondents ranged from Bachelor to Doctorate levels. Then with the measurement algorithm, the reliability and validity of data were checked and then the structural algorithm was used for the measurement of hypotheses of the study. The results obtained from the data were mostly supported. Some of the results were in accordance with previous studies, while some were not. The possible reasons for the difference of results are discussed here. The factor loadings obtained in this study are all above the threshold of 0.5 defined in the literature (Nawaz et al., 2020).

The minimum factor loading of the current study is 0.572 which is well above the threshold. Similarly, regarding the reliabilities, i.e., Cronbach alpha reliability and composite reliability, the cut off value mentioned in literature is 0.7 (Hair and Sarstedt, 2021), however, the obtained reliabilities in this research for the data are above 0.8 which is very good. Furthermore, regarding the average variance extracted, reported in Table 2, the lowest acceptable value given in previous research is 0.5 (Franke and Sarstedt, 2019). The current study meets these criteria of AVE, and the values reported in this study are all above 0.50 which validate the data in this study. The data acquired from the analysis using smart PLS was further validated with hetero trait mono trait, i.e., HTMT ratio, and Fornell and Larcker criterion. For valid data, according to literature, values obtained in the HTMT ratio should be less than 0.9 (Henseler et al., 2015). In this study, the reported values of HTMT ratio are valid making the data valid. Additionally, for Fornell and Larcker criteria, according to Franke and Sarstedt (2019) the topmost values in each column should be among the highest values. Likewise, in our study, the values on the top of the column are among the highest values of each column thus meeting the validity for the study. The results are shown in the form of path models; the direct hypotheses are shown as direct effects while indirect paths show the indirect effects of mediation and moderation. Overall, knowledge hiding and organizational performance have proved to be the major contributors to the proposed conceptual framework of the study. However, if seen individually, organizational performance contributes more to the framework than knowledge-hiding behavior for the current study in the financial services industry in China. This is because although knowledge hiding exists in any organization at every level regardless, the service industry is more prone to it.

There were eight hypotheses developed in this study. The first and the fourth hypotheses suggested that lack of knowledge-sharing reward has an impact on knowledge hiding because when employees are not given due rewards for their efforts in sharing their knowledge they start hiding their knowledge in the future which was supported by the previous studies (Anaza and Nowlin, 2017). Similarly, regarding H_2_ and H_5_, internal competition was also found to have a significant effect on knowledge hiding and organizational performance, respectively, because employees tend to use their knowledge to get an edge over others, and hence hide their knowledge which is also proven in past research (Anaza and Nowlin, 2017). For H_3_ and H_7,_ in this study psychological entitlement was found to have a negative but significant impact on knowledge hiding and then on organizational performance. This is because China has a prevailing socialist market economy where most enterprises are state-owned and parallel the private sector but little ownership exists. Hence, psychological entitlement does not have a positive effect on knowledge-hiding behavior, which contradicts past research (Khalid et al., 2020; Alnaimi and Rjoub, 2021). As far as H_4_ is concerned, knowledge-hiding behavior was found to have a positive and significant effect on organizational performance. This is because when individuals hide knowledge they use their knowledge to get an edge over others that ultimately contributes to organizational performance (Ahbabi et al., 2019). For the last hypothesis of the study that addresses the moderation of the social status of the employee, it was found to have a negative yet significant impact on the relationship of knowledge-hiding behavior and organizational performance because the team member with high social status can interfere with the flow of knowledge (Rhee and Choi, 2017). These results are triggered by the fact that these factors of lack of rewards on knowledge sharing, internal competition, and psychological entitlement are major contributors in knowledge-hiding behavior. Similarly, these all affect the organizational performance of the service industry of financial institutions in China.

## Conclusion

Knowledge hiding is related to decreased work-related interactions of employees, individual performance, and poor decisions (Alnaimi and Rjoub, 2021). More recent literature on knowledge hiding has revealed that the antecedental factors of knowledge hiding regarding individuals, organizations, and teams can enhance their productivity, knowledge sharing, and ultimate performance (Connelly and Zweig, 2015). The major point of focus for this study was also to study the role of antecedents of knowledge hiding at an individual level and how they contribute to the organizational performance. This whole scenario also takes into account the social status of the employees. The current study has found many interesting results regarding the lack of rewards on knowledge sharing; internal competition and psychological entitlement have led to significant contributions to knowledge-hiding behaviors. There have been many opportunities for organizations to determine how they can modify their policies to utilize the potential of employees and their knowledge for the betterment of their organizations. The results obtained in this study have many implications for future studies and policy-makers who are interested in these areas of study for understanding the underlying reasons for knowledge hiding, particularly in the service industry. However, side by side, there are certain limitations of the study. One of the limitations of the study is that this study cannot be replicated in other parts of the world where free economies are practiced. Also, this research can be expanded to other sectors of business and education, which are the biggest hubs for knowledge hiding, where this study can produce more interesting results.

## Data Availability Statement

The original contributions presented in the study are included in the article/supplementary material, further inquiries can be directed to the corresponding author/s.

## Ethics Statement

The studies involving human participants were reviewed and approved by Renmin University of China (RUC), China. The patients/participants provided their written informed consent to participate in this study. The study was conducted in accordance with the Declaration of Helsinki.

## Author Contributions

JW conceived and designed the concept and wrote the manuscript. RM collected the data and helped with analysis. Both authors have read and agreed to the published version of the manuscript.

## Conflict of Interest

JW was employed by company Beijing Docvit Law Firm. The remaining authors declare that the research was conducted in the absence of any commercial or financial relationships that could be construed as a potential conflict of interest.

## Publisher’s Note

All claims expressed in this article are solely those of the authors and do not necessarily represent those of their affiliated organizations, or those of the publisher, the editors and the reviewers. Any product that may be evaluated in this article, or claim that may be made by its manufacturer, is not guaranteed or endorsed by the publisher.
